# Desire thinking as a cognitive mechanism of yearning after bereavement

**DOI:** 10.1016/j.jad.2025.120779

**Published:** 2026-02-15

**Authors:** Hannah Comtesse, Kirsten V. Smith

**Affiliations:** a Department of Psychology, FernUniversität in Hagen (University of Hagen), Hagen, Germany; b Department of Experimental Psychology, University of Oxford, Oxford, UK; c Oxford Health NHS Foundation Trust, UK

**Keywords:** Bereavement, Prolonged grief, Yearning, Craving, Desire thinking, Coping strategies

## Abstract

**Background:**

Yearning is the hallmark of grief and prolonged grief disorder, but its psychological underpinnings are poorly understood. We aimed to address this by building on the phenomenological similarities with craving and investigating the role of desire thinking, a voluntary cognitive process central to activating craving.

**Objective:**

Determining the factorial and psychometric properties of the Oxford Grief Desire Thinking scale (OG-DT) and testing whether desire thinking dimensions prospectively predict proximity seeking, and whether this association is mediated by subsequent yearning.

**Methods:**

We drew on data from the Oxford Grief Study. Two community samples of bereaved adults (cross-sectional *N* = 676, longitudinal *N* = 50) completed the OG-DT and measures of psychological symptoms. A three-wave longitudinal sample (*N* = 275) completed the OG-DT and measures of yearning and proximity seeking at 0–6 months after loss as well as 6 and 12 months later.

**Results:**

The two-factor solution of the OG-DT, imaginal reunion and elaborative efforts, showed excellent internal consistency, test-retest reliability, as well as convergent and criterion validity. Yearning mediated the relationship between both imaginal reunion and elaborative efforts and proximity seeking behaviors.

**Limitations:**

The samples comprised community-dwelling adults, who were predominantly Caucasian and female. Yearning was assessed with a single item.

**Conclusions:**

These findings suggest that desire thinking may be a cognitive driver of yearning, which in turn motivates efforts to cope with the unfulfillable desire. Thus, desire thinking could present a new modifiable target for intervention.

## Highlights

• This is the first investigation of the role of desire thinking in activating yearning after bereavement
• The Oxford Grief Desire Thinking Scale had two factors with (imaginal reunion and elaborative efforts) with excellent psychometric properties
• Yearning prospectively linked early imaginal reunion and elaborative efforts to later proximity-seeking behaviors
• This shows that desire thinking may be a cognitive driver of yearning, which in turn motivates efforts to cope with the unfulfillable desire

## 1 Introduction

The loss of a loved one is one of the most frequently occurring stressful live events (Kessler et al., 2017). Grief is a typical multidimensional response to loss, with yearning being the most common reaction (e.g., Maciejewski et al., 2007; Rosner et al., 2021). Yearning can be described as an intense desire towards a deceased person (Boddez, 2018). Besides its role in grief, yearning is also relevant in psychiatry. It is the hallmark symptom of prolonged grief disorder (PGD), a distressing and disabling grief reaction that persists for a prolonged time after the loss of a loved one (APA, 2022; WHO, 2022). About one in 20 bereaved persons will go onto developing PGD (Comtesse et al., 2024). Research on bereaved community dwellers and treatment seekers has confirmed that yearning is among the most central and prevalent symptoms of PGD (e.g., Malgaroli et al., 2018; Mazza et al., 2025; Robinson et al., 2024; Rosner et al., 2021).

Despite the prominent role of yearning in grief and PGD, there is relatively little research examining its psychological underpinnings. One reason for this may be the lack of conceptual clarity regarding yearning. Clinical observations seem to suggest that it involves physiological and psychological changes from homeostasis. Several fMRI studies have shown that grief-associated stimuli are linked with reward-related activity in bereaved individuals and persons with PGD, indicating considerable similarities between yearning and substance-related craving (Kakarala et al., 2020). Bowlby (1973) defined yearning as a preoccupation with the deceased, motivating proximity seeking of the absent attachment figure, while Carr et al. (2000, p. 200) defined it as “an intermittent, recurrent, and obtrusive wish or need to recover the person who has died”. More recent research aimed at developing a measurement tool for assessing yearning after bereavement and other loss events, the Yearning in Situations of Loss Scale (YSL; Eisma et al., 2020; O'Connor and Sussman, 2014; Robinaugh et al., 2016). In this context, yearning was conceptualized as a cognitive-affective process composed of two components. The cognitive component refers to vividly imagining a counterfactual reality or future in which the deceased is present, while the affective component entails a bittersweet emotional experience related to wanting the person to be with them (e.g., affection and frustration; Eisma et al., 2020). However, the items of the YSL mainly tap into cognitive representations of the desired object, meaning the deceased person, rather than the subjective experience of yearning. Thus, it seems plausible that the YSL assesses a broader concept than yearning alone (see also Robinaugh et al., 2016).

To our knowledge, no studies have yet operationalized the emotional experience of yearning independent of an intentional component of imagining the presence of the deceased. We therefore propose to disentangle these components of yearning by drawing from cognitive models of craving used in addiction research (Caselli and Spada, 2015; Kavanagh et al., 2005, Kavanagh et al., 2009). Here the concept of desire thinking describes an intentional and effortful cognitive process that increases craving for a desired target. Craving bears phenomenological similarities with the experience of yearning (Boddez, 2018) and refers to an intense, subjective experience that motivates one to seek out the target for its rewarding effects (Marlatt, 1987). Desire thinking, in turn, consists of two conscious dimensions: the multisensory elaboration of positive anticipatory imagery or memories related to the desired target (i.e., imaginal prefiguration), and verbal thoughts about good reasons for engaging in target-related activities (i.e., verbal perseveration; Caselli and Spada, 2010). On this basis, we adapted the Desire Thinking Questionnaire (DTQ; Caselli and Spada, 2011) to bereavement to capture desire thinking as a function of imaginal reunion (e.g., *‘I bring to mind the sensations I would feel if we were together’*) and elaborative efforts (e.g., ‘*When I begin to think about [-] I continue until I am exhausted’*) (Smith, 2018) which may act as elaborative intentional precursors of the yearning experience. Importantly, desire thinking differs from other perseverative thinking styles such as rumination in that it involves elaborating positive target-related information.

The cognitive models of craving make specific predictions about how desire thinking enhances and perpetuates craving which in turn motivates behavior (Caselli and Spada, 2015; Kavanagh et al., 2005, Kavanagh et al., 2009). The Elaborated Intrusion Theory (EIT; Kavanagh et al., 2005, Kavanagh et al., 2009) suggests that the duration, frequency, and intensity of craving is the result of the combination of automatic (conditioned) and voluntary cognitive processes, i.e., desire thinking, that are generalizable to all sorts of desires. The target can thus take the form of an activity, object, or state (Kavanagh et al., 2005). According to the EIT, a variety of external and internal triggers lead to the activation of automatic associations that contain information about a desired target. When these associations intrude into awareness (e.g., as intrusive thoughts), they are perceived as spontaneous and induce craving. The escalation and persistence of craving is dependent on the activation of a voluntary cognitive process, i.e., desire thinking, which in turn prolongs and increases the initial craving response (cf. vicious cycle). A growing body of research supports these links between desire thinking, craving, and addictive behaviors across types of addiction (e.g., Mansueto et al., 2019). Recent studies have shown that psychological distress and negative emotions predicted engaging in addictive behaviors through the serial mediation of concurrent desire thinking and craving (Bocci Benucci et al., 2024a, Bocci Benucci et al., 2024b; Brandtner and Brand, 2021; Fioravanti et al., 2024; Olivari et al., 2025). Also, it has been shown that experimentally induced desire thinking evokes craving, regardless of perceived current stress (Caselli et al., 2013).

Against this background, we suggest to map desire thinking onto yearning after bereavement. The loss of a loved person evokes a separation distress response provoked by the attachment system, of which the yearning experience motivates seeking out the absent person (Bowlby, 1973; Shear et al., 2007). Proximity seeking is a coping strategy aimed at restoring closeness to the deceased through reminiscing or memorial activities (cf. continuing bonds; Field et al., 2003) that is used in the acute phase of grief, but also in PGD. These considerations are clinically relevant because we need to identify modifiable factors that are involved in perpetuating yearning due to its central role in grief and PGD. We also need to understand which processes fuel coping strategies such as proximity seeking because they may become maladaptive when they are used to hinder the integration of the loss into autobiographical memory and the correction of negative appraisals of the loss, thus influencing the onset and maintenance of PGD (Boelen et al., 2006; Ehlers and Clark, 2000; Maccallum and Bryant, 2013). However, to our knowledge the mechanisms that specifically drive yearning in bereavement remain unclear.

In this paper, we drew on data from the Oxford Grief Study. Two community sample of bereaved adults were used to examine the factorial and psychometric properties of the Desire Thinking scale for bereavement (OG-DT). In addition, a third three-wave longitudinal sample was used to test whether the desire thinking dimensions of imaginal reunion and elaborative efforts prospectively predicted proximity seeking, and whether this association was mediated by subsequent yearning.

## 2 Methods

### 2.1 Participants and procedure

The study draws on three distinct groups of bereaved adults recruited through bereavement charities, social media campaigns, and the Google content network as part of The Oxford Grief Study. All participants completed symptom questionnaires and the OG-DT online in line with ethical procedures (Smith et al., 2018). They were compensated for their time, provided electronic informed consent, and the studies received approval from the University of Oxford Medical Sciences Inter-Divisional Research Ethics Committee (MS-IDREC-C1–2015-230; MS-IDREC-C1–2015-231). The hypotheses and analyses of the current study were preregistered via *AsPredicted* (see https://aspredicted.org/dtcw-vdfb.pdf). The first dataset was cross-sectional and comprised 676 participants (mean age = 49.22 years, SD = 12.52; 81.5 % women) who had been bereaved for at least 6 months (average time since loss = 56.81 months, SD = 79.79). Within this group, 36.1 % had lost a partner, 28.3 % a parent, 21.0 % a child, 6.5 % a sibling, and 8.2 % another relative or close friend. Nineteen percent of participants reported a violent bereavement. Test-retest reliability of the OG-DT was evaluated in a second sample consisting of 50 individuals bereaved at least 6 months who completed measures twice, one week apart. The average age was 51.46 years (SD = 14.54), with 84 % women. Losses included a parent (42 %), a partner (28 %), a child (22 %), or another relative/close friend (8 %). On average, participants had been bereaved for 23.74 months (SD = 48.44), and 26 % had experienced a violent loss. Finally, to test longitudinal associations over time, a third bereaved sample of 275 adults in their first 6 months of loss were recruited and followed up 6 and 12 months later. Participants were on average 46.43 years old (SD = 13.24) and 75 % female. In this group, 38.2 % had lost a parent, 30.2 % a partner, 8.7 % a child, 5.8 % a sibling, and 17.1 % another relative or close non-relative. Nine percent reported bereavement due to violent circumstances.

### 2.2 Measures

#### 2.2.1 The Oxford Grief Desire Thinking scale (OG-DT)

The original DTQ is a 10- item self-report instrument consisting of two subscales of five items that measures desire thinking in addictive, eating and impulse control disorders measured on a 4-point scale (almost never - almost always) (Caselli and Spada, 2011). The first subscale assesses the perseveration and repetition of verbal thoughts about desire-related content and experience (verbal perseveration). The second subscale measures the tendency to visually imagine desire-related content and experiences (imaginal prefiguration). The DTQ total score and subscale scores have shown good factor structure, internal consistency, test–retest reliability, as well as predictive and discriminative validity (Caselli and Spada, 2011). The adapted version in this study replaced the term ‘desired activity’ with the chosen name of the deceased and dropped the item ‘I imagine myself involved in the desired activity as if it were a movie.’ The final wording of the 9 items were altered with support from psychologists experienced in the treatment of traumatic loss to clarify item content in the context of bereavement.

#### 2.2.2 The Oxford Grief Coping Strategies Scale – Proximity Seeking Subscale (OG-CS-PS)

The Oxford Grief Coping Strategies Scale (OG-CS) is a 23-item self-report tool assessing the frequency of different coping responses following bereavement (Smith et al., 2024). Respondents indicate on a 5-point Likert scale (1 = never to 5 = always) how often they rely on specific strategies. The measure captures four domains: avoidance, proximity seeking, grief rumination, and injustice rumination. The proximity seeking subscale, consisting of seven items, evaluates the degree to which bereaved individuals deliberately engage in strategies aimed at restoring proximity to the deceased (e.g., “I am still carrying out a routine as a way of caring for them”). Reliability was good in the cross-sectional sample (*N* = 676, α = 0.84) and the longitudinal sample (*N* = 275, α = 0.83).

#### 2.2.3 Symptoms of Prolonged Grief Disorder

The Prolonged Grief-13 (Prigerson and Maciejewski, 2008) was used to measure symptoms of severe and enduring grief following bereavement. A modified version of the measure was used, which incorporated six additional items reflecting the diagnostic framework for Persistent Complex Bereavement Disorder included in the DSM section on conditions for further study. From this expanded pool, ten items most consistent with the DSM-5-TR criteria for Prolonged Grief Disorder (American Psychiatric Association, 2022) that map onto the PG-13-R (Prigerson et al., 2021) were retained (for details, see Smith and Ehlers, 2023). Total scores range from 10 to 50. Yearning was measured using item 1 of the PG-13 “How often have you felt a persistent yearning for deceased?” (based on a 5-point scale from ‘never’ to ‘all the time’).

#### 2.2.4 Symptoms of Posttraumatic Stress Disorder

Posttraumatic stress symptoms were measured using the PTSD Checklist for DSM-5 (Weathers et al., 2013). The measure captures the four symptom clusters specified in the DSM-5: intrusive re-experiencing, avoidance, negative changes in mood and cognition, and hyperarousal. It comprises 20 self-report items rated on a 0–4 scale. In this study, participants were asked to complete the scale in reference to the death of their close relative or partner.

#### 2.2.5 Symptoms of Depression

Depressive symptoms were assessed with the Patient Health Questionnaire-9 (Kroenke et al., 2001). This self-report tool contains nine items reflecting DSM-IV-TR criteria for major depressive disorder (American Psychiatric Association, 2000). Respondents indicate how often they have experienced each symptom in the previous two weeks using a 4-point scale from 0 (not at all) to 3 (nearly every day). Scores are summed to produce a total ranging from 0 to 27.

### 2.3 Data analysis

#### 2.3.1 Factor analyses

To evaluate the stability of the measurement model, the cross-sectional sample (*N* = 676) was randomly divided in half (Osborne and Fitzpatrick, 2012). An exploratory factor analysis (EFA) was carried out on the first subsample (*N* = 348) to identify the factor structure, and this structure was then tested on the second subsample (*N* = 328) through confirmatory factor analysis (CFA). As the factors were expected to be correlated, geomin oblique rotation was applied (Muthén and Muthén, 2007). Model evaluation followed multiple criteria: (1) a χ^2^/df ratio below 3 was taken as indicative of acceptable fit; (2) comparative fit index (CFI) values of 0.90 or higher were considered adequate and ≥0.95 as good; and (3) root mean square error of approximation (RMSEA) values ≤0.10 were regarded as acceptable and ≤0.06 as good (Hu and Bentler, 1999; MacCallum et al., 1996). Items were judged to have sufficient factor determinacy when loadings exceeded 0.35. Items with cross-loadings were assigned to the factor that provided the most coherent conceptual fit, and those with weaker loadings were retained on the factor where they loaded most strongly. Modification indices (MIs), representing the improvement in model χ^2^ from freeing correlated residuals, were only considered when the value exceeded 10 and the suggested modification was theoretically plausible (Brown, 2014). As the scale had 4 response points, WLSMV was used as the estimation method where items are treated as ordinal categorical (Kaplan, 2008).

#### 2.3.2 Psychometric validation

Internal consistency and composite reliability were evaluated using McDonald's omega (ω = (Σ|λi|)^2^ / ([Σ|λi|]^2^ + Σδii), where λi represent factor loadings and δii the error variances) applied to the total scale and each factor identified in the EFA (Gadermann et al., 2012). Convergent and criterion validity of the full scale and each factor were assessed by examining correlations with external measures of psychopathology, specifically PGD, PTSD, depression, and proximity seeking as measured by the OG-CS. Average variance extracted (AVE) was calculated for each factor to estimate the proportion of variance in the latent construct explained by its items (Fornell and Larcker, 1981). An AVE of 0.50 or higher was taken as evidence of adequate convergent validity (Hair et al., 2011). Discriminant validity was demonstrated when a factor's AVE exceeded the squared correlations it shared with other constructs, indicating that each subscale captured distinct variance (Henseler et al., 2015). Temporal stability of the scale and its subscales was examined using a test–retest subsample (*N* = 50), with correlations across a one-week interval. Correlations above 0.70 were considered evidence of acceptable reliability over time.

#### 2.3.3 Mediation analyses

A three-wave path model in which factors scores of baseline imaginal reunion (0–6 months post-loss) (see Table 1) each predicted proximity seeking coping strategies at Time 3 (12–18 months) both directly and indirectly through yearning at Time 2 (6–12 months) was specified. Baseline yearning and proximity seeking were entered as autoregressive controls. To avoid misattributing shared variance to the lagged paths, residual covariances among the baseline variables were freely estimated. All parameters were obtained with maximum-likelihood estimation and 5000 percentile bootstrap replications. The same analyses were run for elaborative efforts (Table 1) which were entered as a predictor at baseline. Factor and mediation analyses were conducted using MPlus Version 8 (Muthén and Muthén, 2007).

**Table 1:** Factor analyses of the Oxford Grief Desire Thinking Scale (OG-DT).

		Factors			
		1		2	
Desire thinking items		E/CFA	ESEM	E/CFA	ESEM
1	I imagine myself being with [−] now.	0.83	0.86^⁎⁎⁎^		0.05
2	I imagine what I would feel like if I were with [−] now.	0.99	1.05^⁎⁎⁎^		−0.08
3	I bring to mind the sensations I would feel if we were together.	0.82	0.91^⁎⁎⁎^		0.02
4	When I begin to think about them I find it difficult to stop.		0.02	0.77	0.79^⁎⁎⁎^
5	When I begin to think about them I continue until I am exhausted.		−0.09	0.77	0.87^⁎⁎⁎^
6	I repeat mentally to myself that I want them back.		−0.02	0.99	0.92^⁎⁎⁎^
7	When [−] pops into my mind, I begin to imagine having her/him back with me.		0.37^⁎⁎⁎^	0.83	0.57^⁎⁎⁎^
8	My mind is focused on feeling close to them and I repeat it to try and satisfy it.		0.01	0.88	0.79^⁎⁎⁎^
9	I find myself repeatedly wishing for just one more minute with [−].		0.14	0.84	0.69^⁎⁎⁎^
Correlations of OG-DT factors					
	Factor 1	–	–		
	Factor 2	0.75	0.75	–	–

*Notes:* EFA sample: N = 348, ESEM sample: *N* = 328. Factors labelled as follows: 1 = Imaginal reunion, 2 = Elaborative Efforts. ESEM factor loadings *p* < .05^⁎^*p* < .01^⁎⁎^*p* < .001^⁎⁎⁎^.

## 3 Results

### 3.1 Factorial and psychometric evaluation of the OG-DT

A detailed description of the results of the factorial evaluation of the OG-DT can be found in the Supplementary Material.

### 3.2 Exploratory factor analysis of the OG-DT

In the EFA, inspection of eigenvalues greater than 1 was suggestive of a one-factor solution. However, the second eigenvalue was close to 1 at 0.90. Examination of the scree plot supported a two-factor solution. The fit statistics for the one-factor solution suggested a good fit for CFI = 0.97 but a very poor fit for RMSEA = 0.212 and χ2 = 411.81 on df = 27, χ2:df = 15.25. The fit of the two-factor solution was also very good for CFI = 0.99 but remained poor for RMSEA = 0.126 and χ2 = 114.92 on df = 19.

Given the very large expected parameter change for items 4 and 5 it was decided to model an E/CFA for the two-factor solution with correlated errors between item 4 and 5 to determine whether a viable solution could be reached. Specifying the two-factor solution with a correlated error between item 4 and 5 indicated an excellent fit (CFI = 0.99, RMSEA = 0.056, χ2 = 35.54, on df = 18, χ2:df = 1.97). Factors were labelled as ‘imaginal reunion’ and ‘elaborative efforts.’ All items and standardized factor loadings are presented in Table 1.

### 3.3 Confirmatory factor analysis of the OG-DT

The CFA assessed the fit of chosen two-factor solution with one correlated error using the CFA half of the sample (*N* = 328). The fit statistics for the two-factor model indicated a good fit for CFI = 0.92 but was just above the acceptable threshold for RMSEA = 0.09, and χ2 = 85.19, on df = 25, χ2:df = 3.41. Inspection of the modification indices suggested that a correlated error should be added between the latent factor ‘imaginal reunion’ and item 7 (MI = 44.57). Using an ESEM approach to model a two-factor solution with one correlated error between item 4 and 5 it was found that item 7 loaded significantly on imaginal reunion in the confirmatory sample 168 (0.37, *p* < .001). No other items significantly cross-loaded. The fit statistics for this model were all good (CFI = 0.99, RMSEA = 0.06) or close to good (χ2 = 37.26, on df = 18, χ2:df = 2.07). Table 1 summarizes the standardized factor loadings for the ESEM.

### 3.4 Psychometric validation of the OG-DT

Evidence of total scale and subscale reliability and validity are presented in Table 2. The total scale demonstrated excellent composite reliability and test-retest reliability, suggesting stability of the scores. The scale showed moderate to strong significant correlations with symptoms of PGD, PTSD and depression, confirming criterion validity. Subscales also met assumptions of composite and test-retest reliability, criterion validity, convergent validity, and discriminant validity.

**Table 2:** Psychometric validity of the OG-DT total score and factor scores of imaginal reunion and elaborative efforts.

		Factors		
Reliability/ validity	Measure	OG-DT total	1	2
Composite	McDonald's Omega	0.95	0.96	0.90
Criterion	PG-13 *r*	0.66⁎⁎⁎	0.50⁎⁎⁎	0.68⁎⁎⁎
	PCL-5 *r*	0.61⁎⁎⁎	0.45⁎⁎⁎	0.63⁎⁎⁎
	PHQ-9 *r*	0.53⁎⁎⁎	0.39⁎⁎⁎	0.56⁎⁎⁎
	OG-CS-PS		0.68⁎⁎⁎	0.79⁎⁎⁎
Test-retest	r	0.90⁎⁎⁎	0.81⁎⁎⁎	0.88⁎⁎⁎
Convergent	AVE		0.89	0.61
Discriminate	Inter-construct *r*^2^		0.56	

*Notes:* Factors labelled as follows: 1 = Imaginal reunion, 2 = Elaborative Efforts. *r =* correlation. PG-13 = Prolonged Grief-13 for assessing PGD symptoms. PCL-5 = PTSD Checklist for DSM-5. PHQ-9 = Patient Health Questionnaire-9 for measuring depressive symptoms. OG-CS-PS = The Oxford Grief Coping Strategies Scale – Proximity Seeking. Test-retest reliability confirmed if *r* > 0.70. Convergent validity of factors confirmed if AVE > 0.5. AVE = Average variance extracted. Factorial discriminant validity confirmed if AVE > Inter-construct *r*^2^.

⁎⁎⁎ *p* < .001.

### 3.5 Three-wave mediation model

**Fig. 1:**
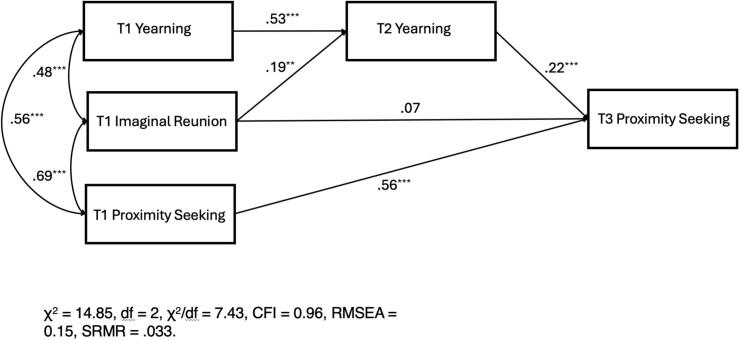
Three-wave mediation model of imaginal reunion predicting proximity-seeking coping strategies via yearning.

**Fig. 2:**
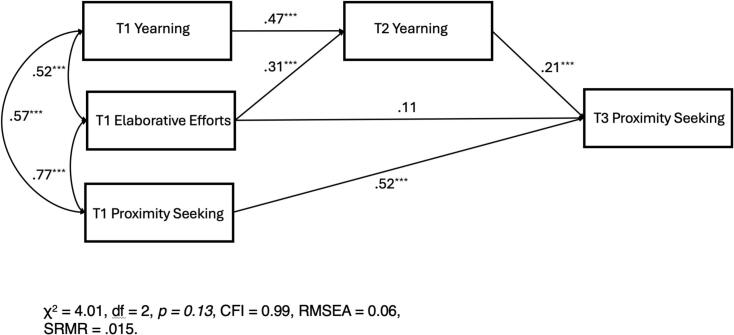
Three-wave mediation model of elaborative efforts predicting proximity-seeking coping strategies via yearning.

Standardized path estimates and model fit results are presented in Fig. 1 for Imaginal Reunion and Fig. 2 for Elaborative efforts.

### 3.6 Model for imaginal reunion

The model was an adequate fit to the data. Although chi-square test was significant, χ^2^(2) = 14.85, *p* = .001, χ2/df = 7.43, incremental and residual fit indices suggested an acceptable representation of the data (CFI = 0.96, TLI = 0.86, SRMR = 0.03). RMSEA was elevated (0.15) but is known to over-reject models with very low degrees of freedom (e.g., Hu and Bentler, 1999); in conjunction with the high CFI and low SRMR we judged overall fit to be adequate.

As hypothesized, higher imaginal-reunion scores at Time 1 predicted stronger yearning six months later, β = 0.19 (SE = 0.06), *p* = .001, 95 % CI = [0.09, 0.31]. Yearning, in turn, was positively associated with subsequent proximity seeking at 12–18 months, β = 0.22 (SE = 0.05), *p* < .001, 95 % CI = [0.12, 0.33]. After yearning and baseline proximity seeking behavior were controlled, the residual direct path from imaginal reunion to proximity seeking was non-significant, *c'* = 0.07 (SE = 0.06), *p* = .29, CI 95 % = [−0.06, 0.20]. The indirect path was significant: β = 0.04 (SE = 0.02), *p* = .005, 95 % CI = [0.03, 0.15]. Thus, imaginal reunion exerted its influence on subsequent proximity seeking entirely through its impact on yearning. The model accounted for 54.9 % of the variance in proximity seeking behaviors.

### 3.7 Model for elaborative efforts

The elaborative efforts mediation model provided a very good fit to the data. The chi-square test was non-significant, χ^2^(2) = 4.01, *p* = .14, yielding a χ^2^/df ratio of 2.00. Complementary indices all pointed to close fit (CFI = 0.99, TLI = 0.98, SRMR = 0.02), and RMSEA was well below the conventional 0.08 threshold (RMSEA = 0.06; 90 % CI = 0.00–0.15).

Consistent with predictions, stronger elaborative efforts at baseline were associated with greater yearning six months later, β = 0.31 (SE = 0.05), *p* < .001, 95 % CI = [0.20, 0.40]. In turn, yearning positively predicted proximity seeking behavior at 12–18 months, β = 0.21 (SE = 0.05), *p* < .001, 95 % CI = [0.11, 0.31]. After accounting for yearning and baseline behavior, the direct path from elaborative efforts to subsequent proximity seeking was non-significant, β = 0.11 (SE = 0.09), *p* = .20 95 % CI = [−0.06, 0.28]. A significant indirect effect emerged, β = 0.07 (SE = 0.02), *p* = .001, 95 % CI = [0.03, 0.11], indicating that elaborative efforts influenced later proximity seeking entirely through their effect on yearning. The model explained 55.6 % of the variance in proximity seeking behavior one year post-loss.

## 4 Discussion

The current study examined the cognitive processes defined in cognitive models of craving (Caselli and Spada, 2015; Kavanagh et al., 2005, Kavanagh et al., 2009), i.e., desire thinking dimensions, in their prediction of the emotional experience of yearning and proximity seeking behaviors in a large bereaved community dataset. The first goal was to determine the psychometric properties of the OG-DT, which was adapted from the DTQ (Caselli and Spada, 2011) to capture desire thinking after bereavement. We found an excellent fit for two factors, imaginal reunion and elaborative efforts, that each showed excellent internal consistency, test-retest reliability, and convergent validity. Both factors demonstrated very good criterion validity, although correlations of elaborative efforts with psychological symptom measures were relatively higher than those of imaginal reunion. It is plausible to assume that the multisensory imagery of being with the deceased (i.e., imaginal reunion) is less distressing or even more comforting than thinking about how to restore closeness to the person to satisfy the deficit the loss has created (i.e., elaborative efforts), which is of course unattainable.

The second aim of this study was to test the function of desire thinking for yearning. As expected, yearning mediated the relationship between both imaginal reunion and elaborative efforts and proximity seeking, even after adjusting for possible confounders of baseline autocorrelations and autoregressions. These results provide strong support the predictions of the EIT (Kavanagh et al., 2005, Kavanagh et al., 2009) and are in line with recent findings from addiction research on the role of desire thinking and craving in evoking addictive behaviors across different domains (Bocci Benucci et al., 2024a, Bocci Benucci et al., 2024b; Brandtner and Brand, 2021; Fioravanti et al., 2024; Olivari et al., 2025). The results demonstrated that desire thinking predicted proximity seeking indirectly via yearning, as the direct effects were not significant in both models. These findings suggest that desire thinking is not directly linked to proximity seeking coping strategies, but instead exerts its influence through heightened yearning. In this sense, desire thinking may be better understood as a cognitive driver that intensifies yearning, which then motivates efforts to remain close to the deceased. This extends research on desire thinking in that it not only shows that its dimensions can prospectively predict yearning, but that this holds even after a long phase of separation from the desired target, i.e., the deceased. Moreover, the results are in line with findings on a moderate concurrent association between proximity seeking and yearning assessed with the YSL (Eisma et al., 2020) in bereaved adults (Eisma and Lenferink, 2023). Yet they point to the usefulness of disentangling the cognitive and affective components of yearning as operationalized through the YSL (Eisma et al., 2020; O'Connor and Sussman, 2014; Robinaugh et al., 2016) in the current study because the emotional experience of yearning emerged as a psychological mechanism linking early desire thinking to later proximity seeking behaviors.

The present results extend our understanding of yearning. We speculate that they map onto a cascade of reactions that builds on the activation of the attachment system (Bowlby, 1973). During life with the now-deceased person, a multitude of internal and external cues and behaviors (e.g., feeling stressed or in need of support, habits, or places) became associated with the person's availability through appetitive conditioning (Boddez, 2018). Following bereavement, yearning is evoked when (conditioned) reminders of the loss (e.g., mental representations of the deceased) intrude into awareness. This experience creates a sense of deficit, which is then imaginatively and verbally elaborated on in an attempt to minimize the distance to the absent person. This intensifies the experience of yearning, which in turn motivates search behaviors aimed at restoring closeness (e.g., dwelling on the deceased's belongings or methods of caretaking). This cascade aligns with the craving processes outlined in the EIT (Kavanagh et al., 2005, Kavanagh et al., 2009) and is supported by findings that PGD symptoms were prospectively linked with proximity seeking (Smith et al., 2024). However, previous research also showed that engaging in proximity seeking behaviors in the first months after the loss predicted grief symptoms later on (Boelen et al., 2006; Field et al., 2003; Smith et al., 2024). It is also plausible to infer the reverse pattern with regard to yearning, meaning a vicious cycle in which proximity seeking prevents yearning from being reduced by blocking the self-correction of thoughts about how to neutralize the distance to the deceased (e.g., ‘I need to stay close’) and preventing the integration of the loss into memory (i.e., upholding the mismatch between the mental representation of the deceased person as someone who is emotionally and physically available and the reality of their absence; Shear et al., 2007). In addition, the current study identified desire thinking as a modifiable factor that could prevent a reduction in yearning. However, this needs to be tested directly in future studies.

Notwithstanding, the current results may have clinical implications. Interventions aim to directly targeting both proximity seeking strategies and desire thinking in order to reduce distress. Proximity seeking is addressed in Internet Cognitive Therapy for Prolonged Grief (iCT-PG) through the use of traditional cognitive therapy methods such as behavioral experiments (Smith et al., 2025). For example, a patient whose young daughter died 25 years earlier still kept her daughter's coat on the household coat rack. The intervention would first identify the negative predictions linked to removing the coat and would operationalize these predictions as observable and testable theories (e.g., ‘I won't be able to remember her face’). The therapist would help the patient recognize any safety behaviors that might prevent learning about the accuracy of these predictions (e.g., moving the coat but putting it back, or holding a photo to avoid “losing” the image) and encourage dropping them during the experiment. The patient could then be invited to recall her daughter's face after moving the coat, and to continue practicing this over several days to evaluate whether the feared outcomes occur. With regard to desire thinking, theoretical models of craving propose that metacognitive beliefs about the usefulness and controllability of desire thinking can activate and interfere with its regulation (Caselli and Spada, 2015). Intervention strategies aimed at increasing flexible control over attention and thinking style were derived from this, utilizing Metacognitive Therapy techniques (Wells, 2009). These techniques have shown promising results for reducing alcohol use (Caselli et al., 2018). In terms of bereavement, a randomized pilot trial has also shown that Metacognitive Therapy could be effective in reducing PGD symptoms and rumination (Wenn et al., 2019).

The results of this study must be considered in light of some limitations. Although our longitudinal design strengthens confidence in the temporal ordering of desire thinking, yearning, and proximity seeking, the analyses do not allow us to establish definitive causal inferences. It remains possible that unmeasured third variables (e.g., overall distress, exposure to external reminders of the loss) may account for part of these associations. Future work using more fine-grained, intensive longitudinal or experimental designs will be important to test causal hypotheses and to identify potential precursors and contextual triggers of yearning (e.g., Kavanagh et al., 2005, Kavanagh et al., 2009). Future studies with larger samples may also be able to model more cognitive processes or coping strategies. For example, we did not investigate the specificity by investigating other perseverative thinking styles such as rumination (e.g., Bocci Benucci et al., 2024a, Bocci Benucci et al., 2024b) that may have influenced yearning. Second, yearning was assessed with a single item. This may have comprised reliability. However, prior research has shown that a single-item assessment of yearning was strongly correlated with the YSL total score, indicating that single items can be an acceptable alternative to assess yearning (Robinaugh et al., 2016). Moreover, the formulation of the yearning item (“[…] felt persistent yearning for the deceased?”) may not have been the best way to assess this emotional experience. It seems to suggest a necessary degree of severity of this experience, which could have led to an underestimation of the amount of yearning in the present study. There were strong correlations between desire thinking and proximity seeking strategies which might point to some multicollinearity in our model. Nonetheless, correlations did not exceed the conventional threshold of 0.80, and while the model specification (i.e., inclusion of baseline residual covariances and autoregressive paths) was designed to mitigate shared variance, some degree of multicollinearity may still remain. Such residual multicollinearity is more likely to inflate standard errors and potentially obscure true effects, suggesting that the mediation observed represents a conservative estimate rather than a spurious one. All data were collected through self-report measures and thus may be affected by typical self-report biases. Finally, the sample was predominately Caucasian and female, which may limit the generalizability of the results. Also, the sample was composed of community dwelling bereaved adults and future research is needed to establish whether the results apply to PGD based on clinical samples.

In conclusion, the current study showed that the experience of yearning links early imaginal reunion and elaborative efforts to later proximity-seeking behaviors. By empirically testing an operationalization of desire thinking after bereavement and investigating predictions derived from cognitive models of craving, this study offers a novel understanding of the cognitive mechanisms of yearning and indicates possible new targets for intervention.

## CRediT authorship contribution statement

**Hannah Comtesse:** Writing – review & editing, Writing – original draft, Methodology, Data curation, Conceptualization. **Kirsten V. Smith:** Writing – review & editing, Writing – original draft, Visualization, Validation, Supervision, Software, Resources, Project administration, Methodology, Investigation, Funding acquisition, Formal analysis, Data curation, Conceptualization.

## Funding

This work was supported by the 10.13039/501100000265Medical Research Council [MR/V001841/1]; the 10.13039/501100000272National Institute for Health Research (NIHR) Biomedical Research Centre, based at Oxford University Hospitals NHS Trust (K. V. Smith), and the NIHR Oxford Health Biomedical Research Centre (K. V. Smith). The views expressed are those of the author(s) and not necessarily those of the NIHR or the Department of Health and Social Care.

## Declaration of competing interest

The authors have no conflicts of interest to declare.

## Data Availability

The ethical approval for this work stipulated that no data could be disseminated outside the research team. The relevant ethics board can be contacted at ethics@medsci.ox.ac.uk for further information. Readers who wish to access the analysis scripts and corresponding outputs may request them from the Centre for Anxiety Disorders and Trauma, University of Oxford, South Parkes Road, Oxford, OX1 3PS (oxcadat.enquiries@psy.ox.ac.uk).

## Appendix A Supplementary data

Supplementary material Image 1
