# Supplementary material for: Desire thinking as a cognitive mechanism of yearning after bereavement
Source: J Affect Disord. 2026 Feb 15;395(Pt B):None. doi: 10.1016/j.jad.2025.120779 (PMC13328055; doi:10.1016/j.jad.2025.120779)
Supplement: Supplementary file 1 — Supplementary material [file mmc1.docx]

**Supplementary Material**

**Factorial evaluation of the Oxford Grief Desire Thinking Scale (OG-DT)**

**Exploratory factor analysis of the OG-DT.** In the EFA, inspection of eigenvalues greater than 1 was suggestive of a one-factor solution. However, the second eigenvalue was close to 1 at .90. Examination of the scree plot supported a two-factor solution. The fit statistics for the one-factor solution suggested a good fit for CFI = .97 but a very poor fit for RMSEA = .212 and χ2 = 411.81 on df = 27, χ2:df = 15.25. Inspection of the modification indices revealed large expected parameter change if the correlations of error terms between three pairs of variables were allowed to vary (i.e., item 1 with item 2; MI = 56.86, item 2 with item 3; MI = 72.32, item 4 with item 5; MI = 100.46). High modification indices for items 1,2, and 3 together suggest that these items would be better accounted for by their own factor and in fact these three items loaded together in the two-factor solution (all items are presented in Table 1). Finally, items 4 and 5 share very similar wording (i.e., ‘when I begin to think about…’) and as such shared variance could be accounted for by wording as well as the latent factor. Failure to investigate and specify shared variance among items appropriately can lead to misspecified models because it assumes that all of the covariation among indicators loading on a given factor is due to that latent dimension and that all measurement error is random (Brown, 2014). Therefore, it makes conceptual sense in this instance to model a two-factor solution with items 1, 2, and 3 loading on the first factor. The second eigenvalue was close to 1 and a parallel analysis of raw data scree plot revealed that a two-factor solution would be appropriate (O’Connor, 2000). The fit of the two-factor solution was also very good for CFI = .99 but remained poor for RMSEA = .126 and χ2 = 114.92 on df = 19.

Given the very large expected parameter change for items 4 and 5 it was decided to model an E/CFA for the two-factor solution with correlated errors between item 4 and 5 to determine whether a viable solution could be reached. An E/CFA strategy applies the same number of identifying restrictions used in EFA by fixing factor variances to unity, by freely estimating the factor covariances, and by selecting an anchor item for each factor whose cross-loadings are fixed to zero while allowing for correlated errors to be modelled, as in CFA. It allows development of a realistic measurement structure prior to moving into the more restrictive CFA framework (Brown et al., 2005). Specifying the two-factor solution with a correlated error between item 4 and 5 indicated an excellent fit (CFI= .99, RMSEA = .056, χ2 = 35.54, on df = 18, χ2:df = 1.97). Factors were labelled as ‘imaginal reunion’ and ‘elaborative efforts.’ All items and standardized factor loadings are presented in Table 1.

**Confirmatory factor analysis of the OG-DT.** The CFA assessed the fit of chosen two-factor solution with one correlated error using the CFA half of the sample (N=328). The fit statistics for the two-factor model indicated a good fit for CFI = .92 but was just above the acceptable threshold for RMSEA = .09, and χ2 = 85.19, on df = 25, χ2:df = 3.41. Inspection of the modification indices suggested that a correlated error should be added between the latent factor ‘imaginal reunion’ and item 7 (MI = 44.57). This cross-loading makes conceptual sense because this item refers to imagining the deceased in present with them as well as the elaborative efforts aimed at restoring proximity. However, this correlated error was not necessary in the EFA sample and in fact the results of the E/CFA showed that this item did not load significantly on imaginal reunion (*p* = .34). To investigate this further an exploratory structural equation model (ESEM) was specified (Asparouhov & Muthén, 2009). ESEM allows for the integration of EFA and CFA measurement models within the same solution by taking into account small cross-loadings that may be well motivated by either substantive theory or by the formulation of the measurements. These small cross-loadings should be interpreted as reflecting the influence of the factor on the construct-relevant part of the item, rather than the item having an impact on the nature of the factor itself (Morin & Maïano, 2011). There is increasing evidence from simulation studies that cross-loadings do not confound the meaning of the latent factors and EFA/ESEM tends to provide more exact estimates of true population values for factor correlations when cross-loadings (even small ones) are present in the population model, and remain unbiased when the population model corresponds to the independent cluster model inherent to CFA (Asparouhov & Muthén, 2009; Howard et al., 2016). Using an ESEM approach to model a two-factor solution with one correlated error between item 4 and 5 it was found that item 7 loaded significantly on imaginal reunion in the confirmatory sample 168 (.37, *p* < .001). No other items significantly cross-loaded. The fit statistics for this model were all good (CFI = .99, RMSEA = .06) or close to good (χ2 = 37.26, on df = 18, χ2:df = 2.07). Table 1 summarizes the standardized factor loadings for the ESEM

**References**

Asparouhov, T., & Muthén, B. O. (2009). Exploratory structural equation modeling. Structural Equation Modeling: A Multidisciplinary Journal, 16(3), 397-438. doi:http://dx.doi.org/10.1080/10705510903008204

Brown, T. A., White, K. S., & Barlow, D. H. (2005). A psychometric reanalysis of the Albany Panic and Phobia Questionnaire. Behaviour Research and Therapy, 43(3), 337-355. doi:10.1016/j.brat.2004.03.004

Howard, J. L., Gagné, M., Morin, A. J., & Forest, J. (2016). Using bifactor exploratory structural equation modeling to test for a continuum structure of motivation. Journal of Management, 0149206316645653. doi:10.1177/0149206316645653

Morin, A. J., & Maïano, C. (2011). Cross-validation of the short form of the physical self-inventory (PSI-S) using exploratory structural equation modeling (ESEM). Psychology of Sport and Exercise, 12(5), 540-554. doi:10.1016/j.psychsport.2011.04.003

O’Connor, B. P. (2000). SPSS and SAS programs for determining the number of components using parallel analysis and Velicer’s MAP test. Behavior Research Methods, Instruments, &amp; Computers, 32(3), 396-402.
